# Technology-enhanced and game based learning for children with special needs: a systematic mapping study

**DOI:** 10.1007/s10209-021-00824-0

**Published:** 2021-07-07

**Authors:** Jose A. Gallud, Monica Carreño, Ricardo Tesoriero, Andrés Sandoval, María D. Lozano, Israel Durán, Victor M. R. Penichet, Rafael Cosio

**Affiliations:** 1 Universidad de Castilla-La Mancha, Campus Universitario s/n, 02071 Albacete, Spain; 2 Universidad Autónoma de Baja California Sur, La Paz, BCS México

**Keywords:** Systematic review, Technology-enhanced learning, Game-based learning, Children with special needs, Primary education, Kindergarten

## Abstract

Technology-based education of children with special needs has become the focus of many research works in recent years. The wide range of different disabilities that are encompassed by the term “special needs”, together with the educational requirements of the children affected, represent an enormous multidisciplinary challenge for the research community. In this article, we present a systematic literature review of technology-enhanced and game-based learning systems and methods applied on children with special needs. The article analyzes the state-of-the-art of the research in this field by selecting a group of primary studies and answering a set of research questions. Although there are some previous systematic reviews, it is still not clear what the best tools, games or academic subjects (with technology-enhanced, game-based learning) are, out of those that have obtained good results with children with special needs. The 18 articles selected (carefully filtered out of 614 contributions) have been used to reveal the most frequent disabilities, the different technologies used in the prototypes, the number of learning subjects, and the kind of learning games used. The article also summarizes research opportunities identified in the primary studies.

## Introduction

Technology-enhanced learning [1] and related educational technologies are an important element in the education system. Moreover, they have become particularly useful during the recent COVID-19 pandemic, by helping to maintain educative activity with the support of online teaching systems.

Children with disabilities form one of the most marginalized and excluded groups in society, whose rights are generally ignored [2]. In recent years, the number of children and young people with disabilities has dramatically increased all over the world.

Previous research articles have presented literature reviews focused on children with special needs [3], inclusive education or technology and inclusion [4]. In [5], the authors present a systematic review of multi-device inclusive environments. Our study is not limited to multi-device systems. Collaborative tools are the research topic of another review [6], which only analyzes contributions from the ACM Digital Library. Some authors have performed a similar study but focused on higher education [7]. In spite of the number of similar literature reviews, key questions such as what the most successful technology applied in the education of children with special needs is, or what learning subjects receive the most attention of researchers, among others, have not yet been answered.

The main goal of this article is to analyze the state-of-the-art of technologies used to support the learning process of people with special needs. The study is focused on educational activities for children in kindergarten and primary education (between 3 and 12 years old).

We start with the definition of a research method, and then define a set of research questions (RQs), performing a comprehensive literature search based on the goal of the research, and answering each research question by showing the results. The systematic mapping presents a group of results in the form of tables and figures. These results can be useful for the research community that works in the field of technology-enhanced and game-based learning applied with children with special needs.

The structure of the article is as follows. Section 2 describes the background and related work. Section 3 defines the research methodology applied in this study, and Sect. 4 contains the answers to the research questions. Section 5 includes a discussion of the results. After the discussion, Sect. 6 analyzes the limitations of this research. Finally, Sect. 7 presents the conclusions and lines for future work.

## Background and related work

This section presents the key terms that are part of the main goal of this study. We have identified three main subsets: technology-enhanced learning, technology applied in the learning process of children with special needs, and game-based learning and gamification in this context.

The term Technology-Enhanced Learning (TEL) refers to the use of technology to enhance the students’ learning process. The foundations and assumptions of technology-enhanced student-centered learning environments are presented in [8].

Information and Communication Technologies (ICTs) is a term employed to stress the role of telecommunications supporting computer software that enable users to access, store, transmit and manipulate information.

ICTs are a key factor when dealing with the learning process of people with special needs due to the advantages of involving psychologists, therapists, traumatologists, neurologists, etc. A review of seven educational technology journals from 1970 to 2011 in which ICTs are used to support learning activities for people with special needs is presented in [9].

The use of TEL in the learning process for people with autism is one of the most widely explored fields. The authors of [10] examine the current state of provision of learning technologies for autistic people and makes recommendations for the design of new technologies and the need for further research. Efforts to enhance the learning process for people with autism are not limited to software design. For instance, the ways in which ICTs are employed to perform technology-enhanced interventions for children with autism can be found in [11].

The assessment of educational activities is a key task to perform during the learning process. A systematic literature review of accessibility recommendations and practices concerning interactive assessment tasks for Science, Technology, Engineering and Mathematics (STEM) is carried out in [12].

Virtual Learning Environments (VLEs) provide a good way of performing learning activities because they can be used in both formal and informal learning scenarios. An analysis of the navigation and exploration of a virtual urban environment comparing autistic children with non-autistic children is presented in [13].

Surveys about how people with special needs interact with computers are also available. The authors of [14] conducted a large-scale survey that collected computer usage information from the parents of approximately six hundred children with Down syndrome.

Serious games have proved to be an effective mechanism for improving learning processes. The potential of serious games as effective and engaging learning resources for people with intellectual disabilities is presented in [15]. In addition, the authors of [16] combine elements of gamification with aspects of accessibility to conceptualize the challenges and possibilities associated with gamified instructional approaches. Gamification models are used in primary education as a learning strategy. In [17], the authors explore the effect of digital games on students’ scientific competencies. In [18], the authors investigate the effectiveness of gamification in teaching numeracy in primary school.

From a technological perspective, the usage of digital games in the learning experience of students with Intellectual Disability (ID) is discussed in [19]. Technologies can be used to assist students with disabilities in accessing the information required to perform the learning process successfully. For instance, AudioLink [20] is an interactive audio-based virtual environment for children with visual disabilities that supports science learning.

Considering these previous works, the experience of applying technologies in schools can be considered positive. However, a recent study [21] shows that technology is used to replicate past teaching practices.

The selection of related works presented in this section shows that (a) there is an actual interest in the research community to explore the possibilities of technology and gamification in the education of children in general and children with special needs in particular, and (b) researchers and practitioners could benefit of studies that analyze how technology is been applied in the learning process of children with special needs.

## Research methodology

This section describes the research methodology applied in this study, which has been adapted from different sources. The backbone has been taken from [22], and the final details have been inspired by [23].

**Fig. 1:**
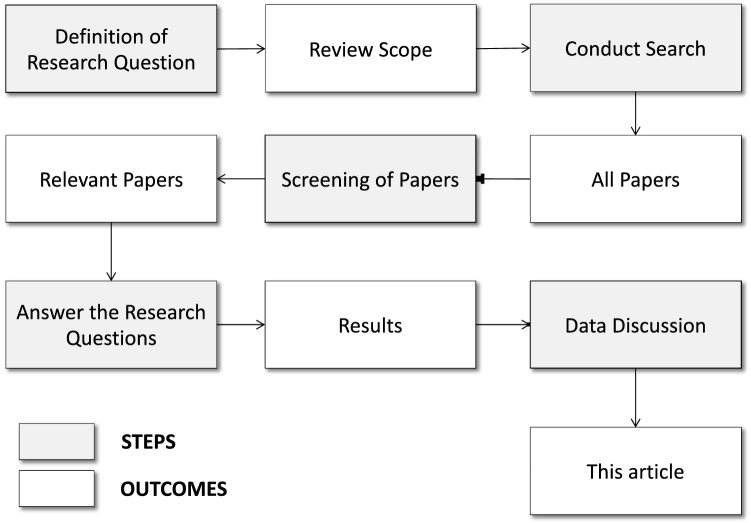
Research methodology used in this systematic review

Figure 1 shows the steps followed in the research methodology, and the main outcomes obtained in each step.

### Definition of research questions

As a first step in the methodology, we defined a set of Research Questions (RQs), which are listed in Table 1 .

**Table 1:** Research questions

**ID**	**Research question**
RQ1	What is the state of the contributions addressing the use of technology to support educational activities for children with special needs published between 2009 and 2019?
RQ1.1	How many academic studies on technology to support educational activities for children with special needs were published between 2009 and 2019?
RQ1.2	What are the publication channels used to publish studies on technology to support children with special needs?
RQ1.3	What is the quality of the selected contributions?
RQ2	What are the disabilities that have been the focus of the primary studies?
RQ3	What are the technologies applied to support educational activities for children with special needs?
RQ4	What kind of learning games are applied in the primary studies?
RQ5	What learning subjects are used in the primary studies?
RQ6	Are the interventions defined in the primary studies effective?
RQ7	What are the research opportunities identified in the primary studies?

### Search conduct

After the definition of the RQs, we conducted several searches on the main research databases using selected keywords related to the topic under study. The search strings used on each database can be found in Table 2.

**Table 2:** Search string used on each database

Database	Search string
ACM digital library	Title:(((“children”OR “student”) AND ((special AND need*) OR “disability”) AND (“education” OR “learning”) AND (“game”))) OR Abstract:(((“children” OR “student”) AND ((special AND need*) OR “disability”) AND (“education” OR “learning”) AND (“game”))) OR Keyword:(((“children” OR “student”) AND ((special AND need*) OR “disability”) AND (“education” OR “learning”) AND (“game”))) “filter”: ACM Pub type: Research article, Publication Date: (01/01/2009 TO 12/31/2019), ACM Content: DL, NOT VirtualContent: true
IEEE xplore	(((“children” OR “student”) AND (“special needs” OR “special need” OR “disability”) AND (“education” OR “learning”) AND (“game”))) Filters Applied: Journals and Conferences 2009 - 2019
ISI web of science	(TS=((“children” OR “student”) AND ((special AND need*) OR “disability”) AND (“education” OR “learning”) AND (“game”))) AND IDIOM: (English) AND TYPE OF DOCUMENT: (Article) Period 2009-2019
ScienceDirect	Title, abstract, keywords: ((“children” OR “student”) AND (“special needs” OR “special need” OR “disability”) AND (“education” OR “learning”) AND (“game”) Filters: 2009-2019 Research articles
Scopus	TITLE-ABS-KEY ( ( “children” OR “student” ) AND ( (special AND need*) OR “disability” ) AND ( “education” OR “learning” ) AND ( “game” ) ) AND ( LIMIT-TO ( DOCTYPE , “cp” ) OR LIMIT-TO ( DOCTYPE , “ar” ) ) AND ( LIMIT-TO ( SUBJAREA , “COMP” ) ) AND ( LIMIT-TO ( PUBYEAR , 2019 ) OR LIMIT-TO ( PUBYEAR , 2018 ) OR LIMIT-TO ( PUBYEAR , 2017 ) OR LIMIT-TO ( PUBYEAR , 2016 ) OR LIMIT-TO ( PUBYEAR , 2015 ) OR LIMIT-TO ( PUBYEAR , 2014 ) OR LIMIT-TO ( PUBYEAR , 2013 ) OR LIMIT-TO ( PUBYEAR , 2012 ) OR LIMIT-TO ( PUBYEAR , 2011 ) OR LIMIT-TO ( PUBYEAR , 2010 ) OR LIMIT-TO ( PUBYEAR , 2009 ) )

Table 3 shows the number of articles found in each database. The total number of articles is 614, including duplicates.

**Table 3:** Number of articles found in each database (2009-2019)

Database	Filter	Papers
ACM digital library	Research articles	14
IEEE xplore	Journal and conference papers	123
ISI web of science	journal and conference papers	168
ScienceDirect	Research articles	15
Scopus	Journal and conference papers	168
**Total**		614

### Screening of papers for inclusion and exclusion

In this step we performed the screening of the papers obtained in the previous steps, to exclude those that are not considered relevant to answer the research questions.

The inclusion criteria were the following:

- The study should be written in English
- The study should be published between 2009 and December 2019
- The study directly answers one or more of the research questions of this study
- The study should clearly state its focus on children with special needs;
- The study should describe the elements and the approach used to implement technology-based systems in primary education.
- If the study has been published in more than one journal or conference, the most recent version is included

Among the exclusion criteria, we applied the following:

- Short papers
- Duplicate articles
- Articles not written in English
- Articles not focused on children with special needs in primary education
- Non-peer-reviewed articles, such as book chapters or technical reports.

**Fig. 2:**
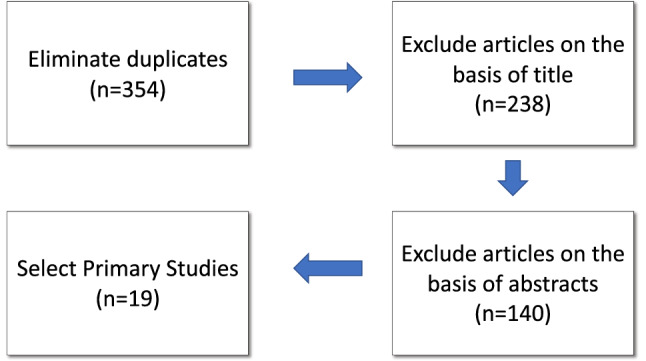
Screening of papers

Some of the criteria will have been successfully applied with the correct definition of the search string, which are included in the previous subsection. The rest of the criteria are applied by following the process described below (see Figure 2), which has been adapted from that of Dybå and Dingsøyr in [24]:

- Stage 1: Eliminate duplicates
  - Download the references (citations in BibTex in our case) from each database
  - Convert the BibTex files into JSON format via scripting
  - Extract relevant information (title, DOI, type of contribution), from each database separately
  - Generate a single list by joining the results obtained from each database and eliminating duplicates taking the DOI as primary key
- Stage 2: Exclude studies on the basis of titles
- Stage 3: Exclude studies on the basis of abstracts
- Stage 4: Select studies by assessing their quality.

After performing the process described in Stage 1, the list of articles without duplicates contained 354 contributions.

The second stage was performed by two groups of two researchers, working separately and comparing the results afterward. The titles of the 354 studies retrieved were read and any titles that clearly indicated that the article was outside the focus of this study were excluded. At the end of this activity 238 papers remained.

In Stage 3, some papers were excluded on the basis of their abstracts. This process was carried out in the same way as in the second stage. The abstracts were revised to exclude articles whose focus was not in line with the main goal of our research (is it applied to children with special needs? does it use technology? is it applied in educational settings?). At the end of this process, 140 articles remained.

The last stage in this phase consisted in assessing the quality of the remaining 140 articles by using a checklist adapted from [24]. This checklist consists of 11 questions about key aspects of the research, method, participants, data collection method, analysis, and findings. At the end of this stage, only 18 works were selected.

One of the important aspects when applying the filtering process is to determine who children with special needs are. A proper answer to this question can be found in [25].

The final collection of articles is the main outcome of the Screening of Papers stage. The complete list of primary studies can be found in 7, where the articles are ordered alphabetically.

## Answering the research questions

Once we have the list of the primary studies, the next step is to answer the research questions.

### RQ1 What is the state of the contributions addressing the use of technology to support educational activities for children with special needs published between 2009 and 2019?

In this section the primary contributions are analyzed to find the number of publications per year (RQ1.1), the main publication channels such as journals or conference proceedings (RQ1.2), and, finally, to identify the quality of the primary contributions (RQ1.3).

### RQ1.1 How many academic studies on technology to support educational activities for children with special needs were published between 2009 and 2019?

**Fig. 3:**
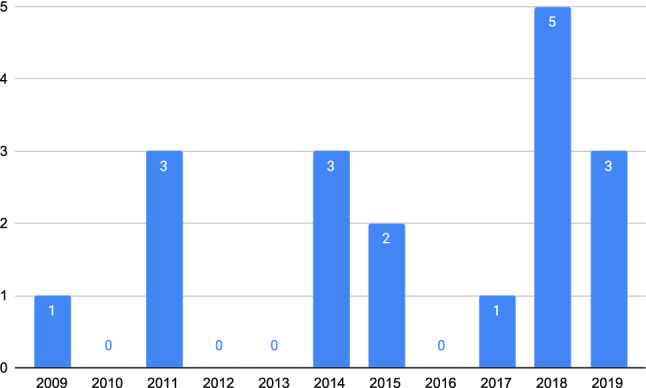
Publications per year (period 2009-2019)

The first research question analyzes the distribution of the primary contributions over the period 2009-2019. Figure 3 shows the number of articles (vertical axis) distributed over the 10-year period (horizontal axis).

The data shown in Figure 3 reveals that there is no particular pattern in the distribution of the primary studies over the period 2009-2019, though over 45% of the primary studies were published in the last two years of this period. On the other hand, the number of related articles (n=354), after the elimination of duplicates, indicates a high degree of interest on this topic on the part of the research community.

### RQ1.2 What are the publication channels used to publish studies on technology to support children with special needs?

This research question summarizes the publication channels of the primary studies. The list of journals and conferences used by the primary studies can be useful to other researchers working on similar topics.

**Table 4:** Primary studies by journal and conference

**Title**	**n**	**PS**
**Channel: Journal (n=10)**		
Universal access in the information society (UAIS)	2	P02, P05
Entertainment computing (EC)	1	P03
International journal of distributed sensor networks (DSN)	1	P04
Journal of applied research in intellectual disabilities (ARID)	1	P07
TechTrends (TT)	1	P08
International journal of environmental research and public health (ERPH)	1	P11
Multimedia tools and applications (MTA)	1	P12
Computer methods and programs in biomedicine (CMPB)	1	P15
Journal of autism and developmental disorders (ADD)	1	P17
International journal of child-computer interaction (CHI)	1	P18
**Channel: Conference (n=7)**		
International conference on serious games and applications for health (SeGAH)	1	P01
IEEE international symposium on multimedia (ISM)	1	P06
European conference on technology enhanced learning (ECTEL)	1	P09
European conference on cognitive ergonomics (ECCE)	1	P10
IFIP conference on human-computer interaction (Interact)	1	P13
International conference on interactive technologies and games (ITG)	1	P14
IEEE games, entertainment, media conference (GEM)	1	P16

Table 4 shows 11 journal articles (corresponding to 10 journals) and 7 conference papers. The list of journals and conferences is diverse and there is only one journal with two primary studies (Universal Access in the Information Society).

A closer look at the list of journals allows us to categorize them according to the main topic of each journal. In the categorization we used the initials of each journal as they appears in Table 4:

- Accessibility (n=1): UAIS;
- Disabilities (n=2): ARID, ADD;
- Human-Computer Interaction (n=1): CHI;
- Health (n=2): ERPH, CMPB;
- Sensors (n=1): DSN;
- Multimedia (n=2): EC, MTA;
- Misc (n=1): TT.

The main topic was selected according to each journal’s title. Some of the journals can be grouped under a more general topic, and some journals share the same topic. For instance, UAIS, ARID, ADD and CHI share an interest in the more general area of Human-Computer Interaction. On the other hand, the general area of Health is included in ARID, ADD, ERPH and CMPB.

There are 8 primary studies that have been published in international conferences (Table 4). The conferences can also be organized according to a main identified topic.

- Games (n=3): SeGAH, ITG and GEM;
- Learning (n=1): ECTEL;
- Human-Computer Interaction (n=1): Interact;
- Multimedia (n=1): ISM;
- Accessibility (n=1): ECCE.

In the conference grouping list we find the term “learning,” which is one of the keywords of this study. Also the term “games” is relevant among the conferences of the primary studies. The field of Human-Computer Interaction represents the main area of the primary studies.

### RQ1.3 What is the quality of the selected contributions?

To answer this research question we define a quality indicator, and to do so we assigned a relevance number, which is based on the PlumX metrics [26]. This indicator will help us to establish a quality metric, which is based on the citation count, the article usage data and the number of captures. To balance these measures, we applied the following formula to obtain a single value (1), where the citations represent 60%, the usage represents 20% and the captures represent 10%.

1 $$\begin{aligned}&relevance=0.60*(cites/MAX\_CITATIONS)\nonumber \\&\quad + 0.20*(usage/MAX\_USAGE)+ \nonumber \\&\quad +0.10*(caption/MAX\_CAPTURE) \end{aligned}$$

Table 5 shows the ordered list of primary studies according to our quality indicator. As expected, the most cited article is the first item in the list according to our quality metric. The three MAX values have been taken from the third stage of the screening (140 articles), where MAX_CITATIONS is 120, MAX_USAGE is 10834 and MAX_CAPTURE is 1447.

**Table 5:** Measuring the quality of the primary studies

PS	Captures	Citations	Usage	Relevance
17	1203	50	6107	0.529
11	208	16	89	0.110
01	66	20	0	0.109
06	98	17	0	0.099
15	261	11	398	0.098
04	302	8	745	0.095
08	117	10	984	0.084
10	111	12	0	0.075
18	132	10	264	0.073
05	54	7	355	0.049
09	49	8	0	0.047
12	88	6	107	0.044
07	85	2	575	0.032
13	26	4	0	0.024
02	64	2	121	0.021
16	22	2	0	0.013
03	55	0	111	0.010
14	52	0	61	0.008

The quality indicator of Equation 1 could take into account those articles published during the last year of the period under study, since the number of citations require some time. In the same way, the data shown in Table 5 represent the relative position of each primary study on the exact date when the data were captured. The three values (citations, captures and usage) increase with time until they reach a top value.

This metric reveals that PS17 can be considered a relevant article with 50 citations, and it is also the reference article in captures and usage. The metric also reveals that there is a considerable distance between PS17 and the group formed by the primary studies 1, 11, 6 and 15, whose relevance is only around 20% of PS17.

### RQ2 What are the disabilities that have been the focus of the primary studies?

**Fig. 4:**
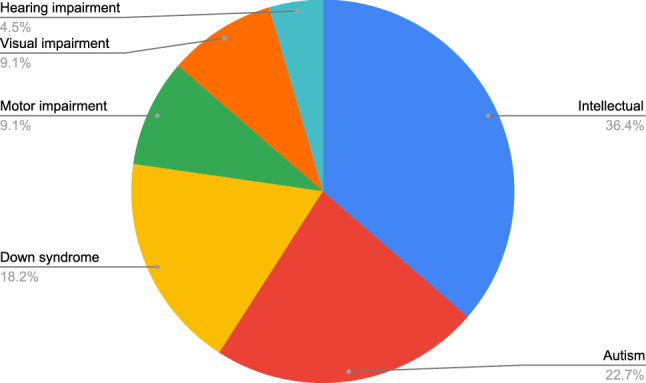
Disabilities researched in the primary studies

This research question analyzes the disabilities that have been the object of research in the primary studies (Figure 4). Some authors prefer to use the term special need condition [6].

It can be observed that 36.4% of the articles focus on intellectual disability, followed by 22.7% for autism and 18.2% for Down syndrome. On the other hand, very few articles focus on visual, hearing and motor disabilities. We could have considered Down syndrome as a specific kind of intellectual disability (ID), which would mean that this type of disability is addressed by up to 54.6% of the articles.

### RQ3 What are the hardware technologies applied to support educational activities for children with special needs?

**Fig. 5:**
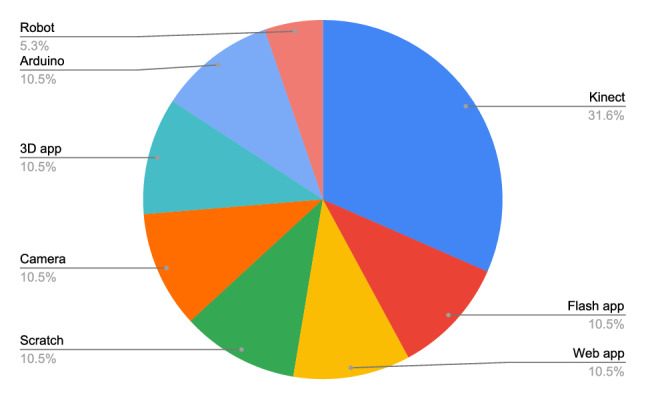
Main hardware technologies employed by the primary studies

The goal of this research question is to find and classify the hardware technologies described in the primary studies. Figure 5 shows the different technologies applied in the primary studies.

Table 6 shows the primary studies grouped by technologies. It can be noted that over 31.5% of the primary studies base their prototypes on Microsoft Kinect [27].

**Table 6:** Technologies and primary studies

Technology	Primary studies
Kinect	PS03, PS07, PS08, PS09, PS12, PS16
Flash	PS01, PS13
Web app	PS02, PS11
Scracth	PS05, PS06
Camera	PS15, PS18
3D app	PS16, PS17
Robot	PS10
Arduino	PS04, PS05

Both, Figure 5 and Table 6 show one main hardware technology as reference in each primary study. However, some primary studies combine the mentioned technology with another hardware and software component. For instance, PS04 uses sensitive resistors and pressure sensors, PS05 uses an electric circuit together with a bracelet, and PS18 also uses a video projector. PS14 is a special case since it applies different educational games without the support of any hardware technology.

### RQ4 What kind of learning games are applied in the primary studies?

This research question summarizes the games used in the prototypes of the primary studies. Table 7 shows the learning game applied in each primary study, together with the associated goal.

Some 50% of the articles are focused on a cognitive therapeutic objective. Articles PS2, PS4 and PS8, focus on games to improve memory.

**Table 7:** Learning games and therapeutic goals in the primary studies

PS	Therapeutic goal	Learning game
01	Cognitive	Shapes
02	Cognitive	Memory, logic sequences
03	Cognitive, social and motor games	Circles, Classifier, Arrows
04	Cognitive	Memory, math, twin match
05	Cognitive	Identify fruits
06	Cognitive	Manage money
07	Social	Soap and water
08	Cognitive	Unboxit, Melody tree
09	Motor	Walks
10	Motor	Cause and effect, imitation game, turn take
11	Cognitive	Manage money
12	Motor, cognitive	Coordination exercise
13	Social	The Natomy’s Journey Game (audio)
14	Social	Magic Potion, storytelling educational game; Street Pirates, a platform game
15	Motor	3D Role Game
16	Motor, Cognitive	Match shapes
17	Social	Recognition game
18	Motor, Social	Circle-Run, Constellation Game

The following list summarizes the therapeutic goals pursued by primary studies: Cognitive (n=9, 50%), Motor (n=6, 33%), and Social (n=5, 27%).

It can be noted that cognitive skills are the main focus of 50% of the primary studies. However, some games classified as cognitive could also be considered as social, as is the case of “Manage money” or “Identify fruits.” Therefore, the distinction between groups is quite subtle.

### RQ5 What learning subjects are used in the primary studies?

The aim of this research question is to find out what are the learning subjects or academic areas that were the focus of the attention in the primary studies (Table 8).

**Table 8:** Learning subject in the primary studies

PS	Learning subject	Area
01	Mathematics skills	Mathematics
02	Cognitive learning	Study Skills
04	Cognitive and learning skills	Study Skills
05	Alternative communication system	Civics Education
06	Concept of money	Civics Education
07	Hand hygiene	Civics Education
08	Short-term memory skills and emotional stage	Study Skills
09	Enhance motor performance	Physical Education
10	Social interaction	Civics Education
11	Money Management Skills	Civics Education
12	Numbers, Shapes, Handwriting	Mathematics
13	Science learning	Natural Sciences
14	Self-determination, engagement and motivation	Leadership
15	Peripheral device	Physical Education
16	Improve both cognition and motor skills	Physical Education
17	Social competence	Civics Education
18	Interpersonal interactions among children	Civics Education

Table 8 shows the learning subject as it appears in each article (column 2). In the third column we have added an Area, which groups similar learning subjects. For example, a set of learning subjects whose main goal is communication, basic hygiene, money management, etc. have been grouped under Civics Education.

There are fuzzy limits between groups. For instance, money management skills has been considered as Civic Education, but it could also have been considered as Mathematics.

### RQ6 Are the interventions defined in the primary studies effective?

This research question has the goal of analyzing to what extend the interventions proposed in the primary studies are effective.

**Table 9:** Research accomplishments in the primary studies

PS	Research accomplishment
01	Participants improved their understanding of fractions.
02	Young people with severe Down Syndrome (DS) may not be motivated enough to use digital games on their own. Young DS people with average concentration ability are engaged by accessible (well-designed) games.
03	The designed video-games combined with embodied interaction, teacher instruction and a turn-taking modality helped the students to train abilities in the motor, cognitive and socio-emotional domains.
04	The system had very positive effects on the children, in terms of cognition and motivational levels.
05	The interactive panel helps children with special needs to achieve learning goals.
06	We have obtained a very positive response by using digital story-telling techniques in their learning process.
07	The parents considered the video game was very useful and it had helped their children learn the hand hygiene skills effectively.
08	Gains in short-term memory ability.
09	Improvements in children’s motor performance, particularly psychomotor ability and psychomotor speed.
10	The interaction with the robots seemed to have in general a positive influence on the development of the children’s social skills.
11	People suffering from DS and other ID respond in a very positive way to the application developed and also to the multi-touch device used.
12	The results showed that the students will be able to use the computer while simultaneously improving their digital competence, and cognitive and physical skills.
13	The results of this study provide initial data and evidence that the use of video games such as The Natomy’s Journey Game can improve school integration process for learners with visual disabilities.
14	Using games as a tool of change and intrinsic motivations, seemed to be a dynamic and promising methodology.
15	It enables people to learn diverse contents, contributing to social and educational inclusion.
16	The results show a great response as both children and their supervisors at the rehabilitation center, where we conducted the test, were happy enjoying the system and showed great enthusiasm for using the game as a training tool.
17	The results demonstrated that the social competence curriculum was delivered with fidelity in the 3D virtual learning environment.
18	It was verified that the visual aid has the capacity to modify students’ running behavior.

Table 9 shows a list of results selected from each primary study. The selection was based on their relevance to the objective of this study. Several primary studies claim their prototypes help children to improve learning (01, 03, 05, 06, 07, 08. 09, 10, 12, 13, 15, 17, and 18). The rest of the contributions indicate their intervention promotes motivation (02, 04, 11, 14, and 16), which is also a result observed in the first group of articles.

### RQ7 What are the research opportunities identified in the primary studies?

The goal of this research question is to enumerate research opportunities in the primary studies (Table 10).

**Table 10:** Research opportunities in the primary studies

PS	Research opportunity
01	Repeat the investigation on a larger sample over a greater number of sessions in the future.
02	Perform additional usability tests with the support of special-needs teachers at school, and with regular training, to investigate the effectiveness of the web platform in supporting cognitive function. Extend the target population to embrace not only the previous sample (children and adolescents) as a follow-up, but also including adults with DS.
03	Future work seeks to deepen the analysis into each described domain, adding new features to the framework allowing the collection data regarding the player’s performance during every game level.
04	Assess the benefits of the edutainment system according to the participants’ knowledge in a normal class to understand whether the system improved learning in other aspects of their education.
05	Include the parents in the system, evaluate the children when playing at home and sharing a natural interaction with their parents; develop low cost technology prototypes for children with special needs to provide both professionals and children with a better interaction and an improved support for learning.
06	Development of more educational games to help patients overcome the social, educational, verbal and behavioral problems.
07	Develop a more technically robust system combined with additional attractive games.
08	Track progressive improvement of skills over time and increase difficulty. Additional use of technologies such as bio-signal sensors, wearable body sensing equipment and EEG (brain waves) and inform a potential practical framework for embodied learning. The enactment of embodied learning using motion-based technology (e.g., Kinect-based educational games).
09	Use clusters of participants with very similar needs to get specific skills clustered. Explore the impact of Kinect-based games on different clusters of participants. Investigate whether any competence developed during the program lasts beyond its duration or even transfers to other domains.
11	Development of new Serious Games to work on other abilities and skills; assess the application orienting it to wider groups of users, such as people without disabilities, and compare the results of different collectives; add a wider variety of coins and notes into the games; and design and develop activities oriented to a real scenario, such as shopping in the supermarket.
12	Create new game configurations to perform new studies.
13	Extend the system to new scenarios providing variations.
14	Validate the results by using a quantitative methodology. Analyze the data trying to observe different relations between parameters regarding learning outcomes, intrinsic motivation and self-determination especially regarding communication and soft skills.
15	Provide access for people with mobility limitations to a serious game to enable learning diverse contents, contributing to social and educational inclusion.
16	Develop more games and levels to cover children with Down syndrome with both cognition and motor skills training needs. In addition, plan to involve game scenarios for teaching children to avoid dangerous items such as sharp tools and fire.
17	Increase the scope of the study.
18	Provide long-term support for students with special needs and realize a model of inclusive education.

As can be observed in Table 10, most of the primary studies propose future research directions related to their presented study (02, 03, 04, 05, 07, 09, 11, 12, 13, and 16). In some cases, the proposal is to increase the sample size (01, 02, 11), the scope (11, 17), or to perform long-term studies (08, 09, 18).

## Discussion

This systematic mapping review has allowed us to select 18 primary studies out of 354 articles. The selected primary studies were focused on the main goal of this study, which is the use of technology-enhanced and game-based learning for children with special needs. We only considered research studies on children in kindergarten, primary and secondary education levels.

Regarding the state of the contributions in this field (RQ1), considering the number of publications in the last 10 years, we can conclude that there is a growing interest in the research community (RQ1.1). The channel of dissemination (RQ1.2) preferred by the primary studies is journals, whose main scope is related to the fields of Human-Computer Interaction, Accessibility, and Health. However, the primary studies belong to a variety of journals, which covers fields ranging from software applications to hardware systems, showing a multidisciplinary nature. The conference papers, according to our quality indicator (based on captures and usage), draw less attention from the readers (RQ1.3).

Research question 2 (RQ2) focuses on the hardware technologies used in the primary studies. The number of prototypes that make use of Kinect [27] is relevant, which can be useful for future works in this field. It is worth to highlight the little use made of visual patterns such as QR-codes. We can also highlight that there is not a single prototype that makes use of NFC/RFID, a technology that is quite popular and widely available in mobile devices. It is striking that there are no prototypes based on tablets or smartphones. Finally, we can also point out the little use made of sensors and, in general, wearable devices.

The next research question (RQ3) analyzes the disabilities addressed in the primary studies. The main interest (77.3%) of the primary studies is focused on intellectual disability, autism and Down syndrome. Some other disabilities have received minor attention (motor, visual and hearing impairment). This fact points to new possibilities for future research. The attention received by autism is noteworthy, and similar results were reported in [6], although in our study, autism is the second disability.

As explained in Sect. 2, in this research we have only considered articles focused on children with a well-defined disability (according to [25]). However, during the screening process, many articles with technology-based proposals to help children with learning problems were excluded.

The research outcomes show the interest of primary studies in intellectual disability (ID). As a consequence, over 50% of the learning games (RQ4) used in the primary studies have cognitive goals, followed by motor and social goals. Some games have been designed to pursue different goals (motor and social, or cognitive and social), which, on the one hand, has the advantage of improving several skills with the same game and, on the other hand, makes it difficult to analyze the effect of the game concerning different conditions.

Among the prototypes that focus on cognitive aspects, games to improve memory and learning geometric figures stand out. To a lesser extent, games to teach money and fruit management also stand out. However, no prototypes of games were found with the aim of learning colors, letters, numbers, mathematical operations, etc. Likewise, no prototypes were found with the aim of learning about nature, the environment or geography.

Research question 5 analyzes learning subjects in the primary studies. According to the results, it is difficult to find works with the aim of improving some of the subjects included in the primary school curriculum, on the contrary, most of them are focused on therapeutic aspects. By analyzing the results, we can see that the main focus is on the area that we have called Civics Education, with 7 primary studies focused on it. Among the traditional subjects only Mathematics and Natural Sciences appear. Another important group of primary studies is focused on cognitive skills. According to these findings, we can conclude that our study has detected a possible gap within this field, namely works that address subjects in the official curriculum in primary and secondary education.

Research question 6 shows a selection of accomplishments extracted from the primary studies. We can point out that some primary studies use conditional sentences to express their findings, which could indicate a non-conclusive result. One of the most common results is that the technology used in each prototype motivates and encourages children with special needs (5 out of 18), which could be considered a good but minor result. Future studies of technology-enhanced learning in children with special needs should focus on improving learning activities and methods, with motivation being a big first step on this road.

The last research question (RQ7) analyzes the research opportunities described by the primary studies. Some articles suggest performing the research on a larger sample, although it is not easy to find a large group of children with special needs. Some articles suggest performing long-term studies (PS02, PS08, PS13, and PS18). PS06 and PS11 highlight the need to develop more educational and serious games. In the primary study 08, the authors point out the opportunity to use bio-signal sensors and wearable devices, aspects that were also mentioned in the discussion of research question 2. Regarding the research method employed, PS14 indicates that future validations should use quantitative methods. This could apply to those articles that base their findings on subjective qualitative methods.

Finally, it is possible to identify promising research lines by crossing the results obtained in this study. For instance, a research line could focus on crossing RQ2 and RQ3 to get a connection between technology (device or software) and type of disability, which could identify the most suitable technology for a particular disability. Another interesting point could be to identify the capabilities needed in future gadgets to be used in special education. We think our study can contribute to this goal by generalizing some of the capabilities that have been successfully applied in children with special needs

## Validity of the study: limitations and threats

Any research work involves a series of validity threats and limitations ([28–30]). In this section, we analyze them and describe the strategies followed to reduce their effects. The validity of this study has been assessed by applying the validity framework presented in [29]. This assessment covers the following aspects: (a) validity of construction, (b) external validity, (c) internal validity, and (d) validity of conclusion.

The *validity of construction* refers to the correctness of the measures used for the concept under study [28] [29] [30]. To reduce this threat, we defined a data collection process to ensure the correct selection of items (e.g., inclusion and exclusion criteria), which was used to filter the contributions according to the criteria defined. To guarantee the coherence of the process and manage this threat, one of the authors was in charge of auditing the protocol throughout the whole process. If any inconsistency was found, the process was repeated from the beginning. The protocol required three iterations to reach the final set of primary studies.

The *external validity* refers to the extent to which the results of the study can be generalized [28] [29]. To know to what extend the results of a study can be generalized, it is extremely important to describe the context of the research [31, 32]. To minimize the impact of this threat, we applied a rigorous research methodology by adapting the guidelines in [22], and we performed the extraction of data (data collection procedures) by following the guidelines in [28] and [24].

The *internal validity* refers to the fact that researchers may not be aware of the connections between the different aspects under study when analyzing causal relations among them. In this study, this has not been a real threat as the different factors under investigation are presented independently and the relationships among them are explicit.

The *validity of conclusion* refers to the influence introduced by the researchers in the analysis of the data. This risk cannot be completely avoided, though it has been reduced by taking the following measures: (a) four researchers participated in the analysis of the primary documents; (b) we conducted a complete audit of the process that filtered 614 documents to identify the 18 primary documents; (c) as stated above, the 140 relevant articles (Stage 4 of the screening) were reviewed by at least two authors, and the conclusions drawn from the analysis of the 18 primary studies were checked by all the authors.

Apart from these four validity threats, we also have to consider the bias in the findings related to the fact that positive research results are more likely to be published than negative results [33]. In this study, this kind of bias has a minimal effect as the objective of the study is to present the outcomes of a systematic review of technology-enhanced and game-based research applied on children with special needs in primary education. However, we recognize that the publication bias could have affected our results with respect to the benefits and challenges of using technology with children with special needs.

We have limited the research study to the period 2009-2019, which could also be considered a limitation as we have not included the most recent contributions in this field published in 2020.

The data sources and their publication channel may also produce bias in the outcomes. In this work we have used the following research databases: ACM digital library, IEEE Xplore, ISI Web of Science, Science Direct and Scopus, since it is well known that these sources contain most of publications and have been used in similar approaches in literature reviews in areas such as software engineering ([23, 34]).

Other types of research works such as scientific studies, short articles, experience reports and assimilation studies, which are not peer-reviewed, were excluded, as they usually present work in progress or preliminary studies whose relevance in the field is considered low.

There is an alternative method proposed in [35] for conducting Systematic Literature Reviews that could be worth exploring.

## Conclusions and future work

This systematic mapping study of technology-enhanced and game-based learning for children with special needs is based on a selection of 18 articles out of a total of 354 papers published during the period 2009-2019. The primary studies are distributed over the period, although the last two years account for almost 50%, which shows the current and increasing interest of the research community in this field.

Among the findings, we can highlight the considerable importance given to Intellectual Disability (ID), Autistic Spectrum Disorder (ASD), and Down Syndrome by the primary studies. As far as technological devices are concerned, the Microsoft Kinect device is the most commonly used hardware platform, followed by different software apps. Over 45% of the prototypes found in the primary studies are focused on improving cognitive skills. Improving social skills in children with special needs is also relevant. Regarding the learning subject, it can be observed that Mathematics (numbers, geometric shapes, money management) and Civic Education are the most frequent academic areas of interest. The article also enumerates a list of research opportunities that could be the seed for future research works.

The research method includes a thorough analysis of the limitations of the research method applied. For instance, the study shows the relative relevance of the primary studies among the selected group of contributions from the screening (n=140 in Stage 3), which is used to measure the quality of the contributions.

A systematic review is a research activity that should promote and inspire new lines of research. In the course of this article, different areas to explore in future works have been pointed out, such as the disability under consideration, the most common hardware devices used in prototypes, the academic subject that should be improved, or the learning game used in the research. All these aspects offer new research possibilities, some of which can be found in the corresponding section of this article.

## Primary studies

1. Brown D. J., Ley J., Evett L., and Standen P. 2011. Can participating in games based learning improve mathematic skills in students with intellectual disabilities? IEEE 1st International Conference on Serious Games and Applications for Health (SeGAH): 1-9.
2. Buzzi M.C., Buzzi M., Perrone E., and Senette C. 2019. Personalized technology-enhanced training for people with cognitive impairment. Universal Access in the Information Society, 18(4):891-907. attention deficit hyperactivity disorder. International Journal of Human-Computer Studies, 126:26-43.
3. Contreras M. I., Garcia, C., and Santos G. 2019. Videogame-based tool for learning in the motor, cognitive and socio-emotional domains for children with Intellectual Disability. Entertainment Computing, 30:3-13.
4. Dandashi A., Karkar A.G., Saad S., Barhoumi Z., Al-Jaam J., and El Saddik A. 2015. Enhancing the cognitive and learning skills of children with intellectual disability through physical activity and edutainment games. International Journal of Distributed Sensor Networks, 11(6):1-11.
5. Durango I., Carrascosa A., Gallud J. A., and Penichet V. M. R. 2018. Interactive fruit panel (IFP): a tangible serious game for children with special needs to learn an alternative communication system. Universal Access in the Information Society, 17(1):51-65.
6. Hassan A. Z., Zahed B. T., Zohora F. T., Moosa J. M., Salam T., Rahman M. M., Ferdous H. S., and Ahmed S. I. 2011. Developing the Concept of Money by Interactive Computer Games for Autistic Children. IEEE International Symposium on Multimedia: 559-564.
7. Kang Y-S., and Chang Y-J. 2019. Using a motion-controlled game to teach four elementary school children with intellectual disabilities to improve hand hygiene. Journal of Applied Research in Intellectual Disabilities, 32(4):942-951.
8. Kosmas P., Ioannou A., and Retalis S. 2018. Moving Bodies to Moving Minds: A Study of the Use of Motion-Based Games in Special Education. TechTrends, 62(6):594-601.
9. Kosmas P., Ioannou A., and Retalis S. 2017. Using embodied learning technology to advance motor performance of children with special educational needs and motor impairments. European Conference on Technology Enhanced Learning, 10474 LNCS:111-124.
10. Lehmann H., Iacono I., Robins B., Marti P., and Dautenhahn K. 2011. ’Make it move’: Playing cause and effect games with a robot companion for children with cognitive disabilities European Conference on Cognitive Ergonomics: 105-112.
11. Lopez-Basterretxea A., Mendez-Zorrilla A., and Garcia-Zapirain B. 2014. A Telemonitoring Tool based on Serious Games Addressing Money Management Skills for People with Intellectual Disability. Journal of Environmental Research and Public Health, 11(3):2361-2380.
12. Ojeda-Castelo J. J., Piedra-Fernandez J. A., Iribarne L., and Bernal-Bravo C. 2018. KiNEEt: application for learning and rehabilitation in special educational needs. Multimedia Tools and Applications, 77(18):24013-24039.
13. Sánchez J., and Sáenz M. 2009. Video gaming for blind learners school integration in science classes. IFIP Conference on Human-Computer Interaction, 5726 LNCS:36-49.
14. Saridaki M., and Mourlas C. 2014. Flow, Fun and Frame in the Classroom: Redefining the Engagement and Self-Determination of Students with Intellectual Disability through Games. International Conference on Interactive Technologies and Games: 44-50.
15. Scardovelli T.A., and Frère A.F. 2015. The design and evaluation of a peripheral device for use with a computer game intended for children with motor disabilities. Computer Methods and Programs in Biomedicine, 118(1):44-58.
16. Shalash W.M., Altamimi S., Abdu E., and Barom A. 2018. No Limit: A Down Syndrome Children Educational Game. IEEE Games, Entertainment, Media Conference: 352-358.
17. Stichter J. P., Laffey J., Galyen K., and Herzog M. 2014. iSocial: Delivering the Social Competence Intervention for Adolescents (SCI-A) in a 3D Virtual Learning Environment for Youth with High Functioning Autism. Journal of Autism and Developmental Disorders, 44(2,SI):417-430.
18. Takahashi I., Oki M., Bourreau B., Kitahara I., and Suzuki K. 2018. FUTUREGYM: A gymnasium with interactive floor projection for children with special needs. International Journal of Child-Computer Interaction, 15:37-47.
